# Predicting Factors Associated with Hypoglycemia Reduction with Automated Predictive Insulin Suspension in Patients at High Risk of Severe Hypoglycemia: An Analysis from the SMILE Randomized Trial

**DOI:** 10.1089/dia.2019.0495

**Published:** 2020-09-03

**Authors:** Aklilu Habteab, Javier Castañeda, Harold de Valk, Pratik Choudhary, Emanuele Bosi, Sandrine Lablanche, Simona de Portu, Julien Da Silva, Linda Vorrink-de Groot, John Shin, Ohad Cohen

**Affiliations:** ^1^ Medtronic Bakken Research Center, Maastricht, The Netherlands.; ^2^ Department of Internal Medicine, University Medical Center, Utrecht, The Netherlands.; ^3^ Department of Internal Medicine King's College Hospital, Diabetes Research Group, London, United Kingdom.; ^4^ Diabetes Research Institute, IRCCS San Raffaele Hospital and San Raffaele Vita Salute University, Milano, Italy.; ^5^ Department of Diabetology, Grenoble University Hospital, Service d'Endocrinologie, Grenoble, France.; ^6^ Medtronic International Trading Sàrl, Tolochenaz, Switzerland.; ^7^ Medtronic, Northridge, California, USA.

**Keywords:** Diabetes mellitus, Type 1, Hypoglycemia, Insulin infusion systems, Severe hypoglycemia, Predictive low glucose management

## Abstract

***Background:*** This analysis from the SMILE randomized study was performed to identify predictive factors associated with the greatest reductions in hypoglycemia with the Medtronic MiniMed™ 640G Suspend before low feature in adults with type 1 diabetes at high risk of severe hypoglycemia.

***Methods:*** Clinical and treatment-related factors associated with decreased sensor hypoglycemia (SH) were identified in participants from the intervention arm by univariate and multivariate analyses.

***Results:*** The reduction in SH events <54 mg/dL (<3.0 mmol/L) in the intervention group was significantly (*P* < 0.0001) associated with the baseline mean number of sensor hypoglycemic events (MNSHE) <54 mg/dL. When excluding continuous glucose monitoring (CGM) factors not readily available (MNSHE, duration of SH events, area under the curve, mean amplitude of glycemic excursions), only the baseline mean time spent <54 mg/dL was found to be a significant independent predictor factor (*P* < 0.0001). Baseline HbA1_c_, mean self-monitoring of blood glucose (SMBG), and coefficient of variation of SMBG were significant, although weak, predictors in the absence of any CGM data.

***Conclusions:*** The greatest reductions in SH events achieved with the MiniMed 640G system with the Suspend before low feature were seen in participants with higher baseline MNSHE. Measuring these (usually uncollected) events can be a useful tool to predict hypoglycemia reduction. ClinicalTrials.gov Registration Identifier NCT02733991.

## Introduction

Attempts to achieve optimal glycemia often incur an increased risk of severe hypoglycemia.^1-3^ Risk factors for severe hypoglycemia include duration of diabetes, previous severe hypoglycemic events, and hypoglycemia unawareness, which are present in ∼20% of people with type 1 diabetes.^4,5^ The recent SMILE (Study of MiniMed 640G insulin pump with Smart Guard in prevention of low glucose events in adults with type 1 diabetes) study^6,7^ showed that the use of the Medtronic MiniMed™ 640G insulin pump system with continuous glucose monitoring (CGM) and automated predictive low glucose management (PLGM) reduced hypoglycemic episodes (defined as sensor glucose ≤55 mg/dL [3.1 mmol/L] lasting ≥20 consecutive minutes) by 73%, and severe hypoglycemic episodes by 83.3%, in adults with type 1 diabetes at high risk of severe hypoglycemia.

This further analysis of the SMILE study data examined the association between the reduction in hypoglycemic episodes during the study and baseline characteristics in participants in the intervention arm, to identify factors predictive of greater reductions in hypoglycemia, and hence those individuals most likely to benefit from PLGM suspend before low functionality.

## Materials and Methods

The methods of the SMILE study have been previously described.^6,7^ Briefly, this was a 6-month randomized controlled trial involving 169 participants aged 24–75 years with a ≥10-year duration of type 1 diabetes. After a 2-week run-in phase with blinded CGM, participants who successfully completed this phase were randomized to either the MiniMed 640G system (Medtronic, Northridge, CA, USA) with CGM and the SmartGuard™ PLGM suspend before low feature continuously turned on (intervention), or the MiniMed 640G pump without CGM (control), for 24 weeks.

### Statistical analyses

Data from participants in the intervention arm were used to assess the association between baseline demographic and clinical factors and the reduction in mean number of sensor hypoglycemic events (MNSHE) during the study phase. The baseline demographic and clinical factors listed in Supplementary Table S2 were identified as clinically relevant, and they were considered for analysis. A sensor hypoglycemic event was defined as a sensor glucose <54 mg/dL for ≥20 consecutive minutes. Two-week CGM data collected during the run-in and on three occasions beginning at weeks 10, 16, and 22 of the study phase (period 1, 2, and 3, respectively) were used. The MNSHE during the study phase was calculated as the mean of the three periods, and the reduction in MNSHE was calculated as the change from baseline to study phase. For all CGM factors, data were considered to be missing in any period when sensor data were available for less than 3 days; no imputation of missing data was performed.

Statistical analysis of associations between baseline factors and reductions in hypoglycemia consisted of univariate analysis followed by multivariate analysis.

Univariate analysis with MNSHE change as response variable was first conducted for each variable separately: Factors with *P*-values <0.2 were retained for further analysis, and correlations between these factors were assessed by using Pearson correlation. Although the Pearson correlation was >0.7 or <-0.7, only the factor with the smallest *P*-value in univariate analysis was included in the multivariate analysis, to avoid multicollinearity.

Three multivariate analyses were performed. Model 1 (the primary analysis) considered all baseline demographic, CGM, and clinical factors; Model 2 excluded CGM factors that are not readily available with current commercial CGM systems (MNSHE, duration of sensor hypoglycemia event, area under the curve, and MAGE); and Model 3 excluded all CGM-related factors, to identify factors predictive of hypoglycemia reduction in individuals not using real-time CGM.

For each of these models, a saturated model was used initially, including all the potential predictive factors identified in univariate analysis, and the final model was selected by manual backward selection; the most insignificant variables were eliminated one at a time until a parsimonious model with significant factors (*P* < 0.05) was identified.

Because the selected model included only participants in the intervention arm of the study, a sensitivity analysis including all randomized participants was performed by using the final model, with interaction with study arm (intervention or control) as a factor. Significance of the interaction in this sensitivity analysis was taken as confirmation that significance in the primary analysis was not due solely to a possible Hawthorne effect (i.e., the results were not attributable to study participation per se).

## Results

The baseline characteristics of subjects randomized to the MiniMed 640G system with suspend before low feature have been previously published.^7^ The present analysis included 70 participants from the intervention arm: 6 participants in this arm had fewer than 3 days of CGM data at baseline, and they were excluded from this analysis. The baseline characteristics of the included participants were comparable to the entire study population in the intervention arm (*n* = 76) (Supplementary Table S1).

Supplementary Table S2 lists the factors that were significantly (*P* < 0.2) associated with reductions in MNSHE <54 mg/dL in univariate analysis. The final multivariate models are shown in Table 1.

**Table 1.** Final Multivariate Models of the Association of Baseline Factors and Reduction of Mean Number of Sensor Hypoglycemic Event Per Week in Participants Using the MiniMed 640G with Suspend Before Low Feature Turned On

	Regression coefficient	Standard error	*P*
Model 1 (primary analysis): all baseline demographic and clinical factors included			
Baseline MNSHE	−0.7937	0.029	<0.0001
Model 2: CGM factors that are not readily available with current commercial CGM systems excluded^a^			
Baseline time (minutes) spent <54 mg/dL	−0.0565	0.002	<0.0001
Model 3: all CGM-related factors excluded			
Baseline HbA_1c_	0.6826	0.33	0.04
Baseline mean SMBG	0.0341	0.01	0.002
Baseline coefficient of variation of SMBG	−13.5020	3.57	0.0003

^a^ MNSHE, duration of MNSHE, AUC, and MAGE.

AUC, area under the concentration-time curve; CGM, continuous glucose monitoring; MAGE, mean amplitude of glycemic excursions; MNSHE, mean number of sensor hypoglycemic events; SMBG, self-monitoring of blood glucose.

**FIG. 1.**
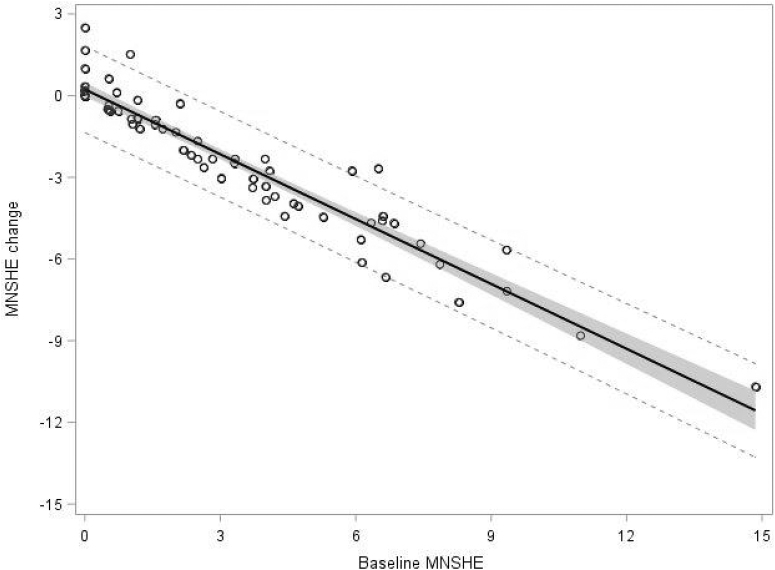
Change in MNSHE per week by baseline MNSHE per week. Solid line: regression line based on Model 1. Shaded area: 95% confidence interval for the regression line. Dotted lines: upper and lower limit of the 95% prediction interval. MNSHE, mean number of sensor hypoglycemic event.

In model 1, baseline MNSHE <54 mg/dL was the only baseline factor found to be significantly (*P* < 0.0001) associated with a reduction of MNSHE <54 mg/dL. The model *mean reduction of MNSHE = 0.2271 − 0.7937 × (baseline MNSHE)* explained 91.3% of all variability. Participants with higher MNSHE at baseline showed larger reductions in MNSHE (Fig. 1); in a participant with a baseline MNSHE of 4, MNSHE would be reduced on average to 1.05 (73.7% reduction in sensor hypoglycemic events) (see Supplementary Table S3 for different baselines). Analysis for events based on 60 and 70 mg/dL was also performed as a sensitivity analysis, with similar results (Supplementary Table S4). Baseline MNSHE <60 and <70 mg/dL were the only significant factors associated with the reduction of MNSHE <60 mg/dL (*P* < 0.0001, *R*^2^ = 83.9%) and <70 mg/dL (*P* < 0.0001, *R*^2^ = 71.2%).

In model 2, baseline time spent below 54 mg/dL was the only significant factor (*P* < 0.0001) and 85.1% of the variability was explained by the model, *mean reduction of MNSHE = 0.0862 − 0.0565 × (baseline time spent below 54 mg/dL)*. Participants who spent more time below 54 mg/dL at baseline experienced greater reductions in MNSHE (Supplementary Fig. S1): Each 10 min/day spent below this threshold at baseline was associated with an additional reduction in MNSHE of 0.57.

In model 3, baseline mean SMBG, coefficient of variation (CV) of SMBG, and HbA1c were significant, but this model [*mean reduction of MNSHE = −6.9297 + 0.6826 × (baseline HbA1c) +0.0341 × (baseline mean SMBG) −13.5020 × (baseline CV of SMBG)*] explained only 38.7% of all variability.

When an interaction between study arm and baseline MNSHE was included in Model 1 and fitted to data from all randomized participants, the interaction term was significant (estimate = 0.3848, *P* = 0.0007): A larger reduction in MNSHE was seen in the intervention arm than in the control arm (Supplementary Fig. S2). This confirms that the significant results seen in the primary analysis are not attributable to a study effect.

## Discussion

The analysis reported here extends the findings of the SMILE study,^7^ showing that baseline MNSHE is the strongest predictor of reductions in MNSHE; each additional MNSHE <54 mg/dL at baseline was associated with an additional 0.8 reduction in MNSHE. This measure consistently predicted reduction in hypoglycemia events, irrespective of the MNSHE threshold (70, 60 or 54 mg/dL). However, the 54 mg/dL threshold demonstrated the highest attributable prediction (*R*^2^ = 0.913), showing that PLGM provides the most robust protection from hypoglycemia in the most severe situation.

The finding that the extent of hypoglycemia reduction induced by technological interventions is a function of the baseline prevalence of hypoglycemia is not new: A 2008 meta-analysis of 22 studies in people with type 1 diabetes found that the greatest reductions in hypoglycemia achieved with continuous subcutaneous insulin infusion were seen in individuals with the highest rates of severe hypoglycemia on multiple daily injections.^8^ Rather, the novelty of the present finding lies in the magnitude of the effect, and the fact that it remained independent and significant in multivariate analysis; whereas other factors associated with hypoglycemia in univariate analysis, such as insulin dose, age and duration of diabetes, HbA_1c_, mean sensor glucose, and other CGM measures, lost significance when modeled with baseline MNSHE. The phenomenon of regression to the mean in MNSHE can be seen in the control arm: There was no overall change in MNSHE from baseline to study phase, but participants with higher or lower baseline MNSHE showed reductions or increases in MNSHE, respectively, during the study phase. By contrast, in the intervention arm, there was a systematic reduction in MNSHE that was both statistically and clinically significant (Supplementary Fig. S2).

Because measurement of hypoglycemic events, as used in the SMILE study, is not readily available with currently available commercial CGM systems, we modeled the different factors excluding baseline MNSHE in Model 2. Among more accessible CGM-derived factors, only the baseline time spent below 54 mg/dL was found to be a significant and independent predictor (*P* < 0.0001; Supplementary Table S2). This factor explained 85.1% of the variability in MNSHE reduction in relation to baseline MNSHE. Lastly, when assessing all clinical and demographic factors available at baseline, excluding CGM-derived data (Model 3), HbA_1c_, mean SMBG, and CV of SMBG were statistically significant predictors, but their cumulative contribution to the variability of hypoglycemic episode reduction was smaller (38.7%).

From a clinical perspective, these findings suggest that baseline MNSHE is the strongest predictor of hypoglycemia reduction, and it should be considered an important metric; time below 54 mg/dL can be used when MNSHE is not readily available. Without CGM data, baseline HbA_1c_, mean SMBG, and CV of SMBG have a poor predictive value (38.7%). Therefore, CGM data are required to identify responders to therapy.

Interestingly, in the present analysis, age, gender, and body mass index were not associated with hypoglycemia reduction. Similarly, weight-corrected total daily insulin dose and total daily bolus dose were associated with hypoglycemia reduction on univariate analysis, whereas the type of insulin and the mean number of boluses per day were not. The Clarke score was also not predictive of hypoglycemia reduction: This may be because the included participants were hypoglycemia unaware, as defined by a Clarke score of ≥4 or Gold score ≥4, and hence little variability in Clarke score would be anticipated in this population.

The definition of a hypoglycemic episode as a sensor glucose below 54 mg/dL followed guideline recommendations,^9,10^ and the duration of ≥20 consecutive minutes has been used in previous studies.^11^ Because the Guardian Sensor 3 reports the glucose value every 5 min, a hypoglycemic event consists of at least four sensor glucose measurements, and hence can be considered a true episode of hypoglycemia. The SMILE study provided additional validity to this definition, by demonstrating a reduction in severe hypoglycemic events, which are clinical entities with clinical implications. By contrast, the recent consensus defining hypoglycemia as a minimum 15-min interval without consideration of sampling number^12^ is not evidence based.

A strength of this analysis is that the data were collected from a robust, prospective study, across a number of sites and countries, with a relatively long follow-up, which provided evidence of significant reductions of severe hypoglycemia in the intervention arm. Potential limitations are the post hoc nature of the analysis, and the fact that results are applicable only to the SMILE study population of people with type 1 diabetes and high risk of hypoglycemia, and hence the findings may not be generalizable to the wider diabetic population. In addition, the analysis used only 6 weeks of data, rather than the data from the full 6-month study. This approach was adopted for consistency with the main publication results,^7^ and to allow comparison with the regression seen during the three CGM periods in the control arm.

In conclusion, this analysis shows that the significant reduction of sensor hypoglycemic events demonstrated in the SMILE study with the MiniMed 640G system with the Suspend before low feature is strongly associated with the baseline MNSHEs. Measuring these events with CGM can serve as an important tool to predict hypoglycemia reduction, and for appropriate therapy selection. If this measurement is not available, time spent in hypoglycemia could be a valuable alternative, which is easier to implement in clinical practice.

## Supplementary Material

Supplemental data
