# Supplementary material for: Predicting Factors Associated with Hypoglycemia Reduction with Automated Predictive Insulin Suspension in Patients at High Risk of Severe Hypoglycemia: An Analysis from the SMILE Randomized Trial
Source: Diabetes Technol Ther. 2020 Sep 3;22(9):681–5. doi: 10.1089/dia.2019.0495 (PMC7478192; doi:10.1089/dia.2019.0495)
Supplement: Supplemental data [file Supp_Data.pdf]

# Supplementary Data

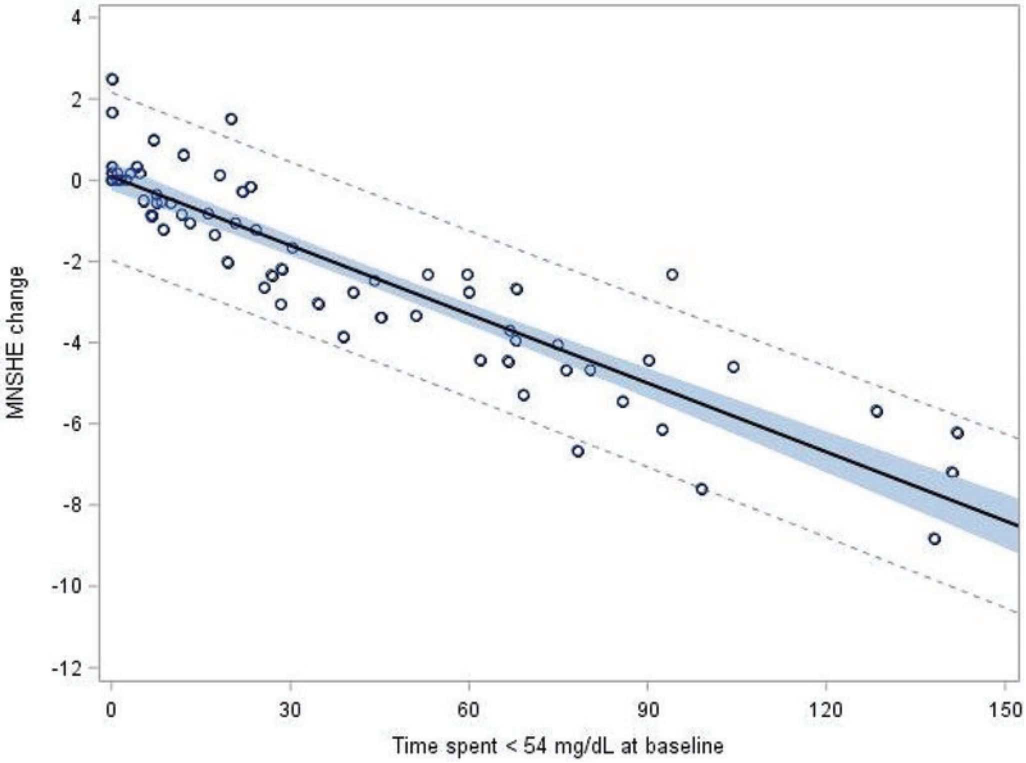


**SUPPLEMENTARY FIG. S1.** Change in MNSHE per week by baseline time spent below 54 mg/dL in minutes per day. Solid line: regression line based on Model 2. Shaded area: 95% confidence interval for the regression line. Dotted lines: upper and lower limit of the 95% prediction interval. MNSHE, mean number of sensor hypoglycemic events.

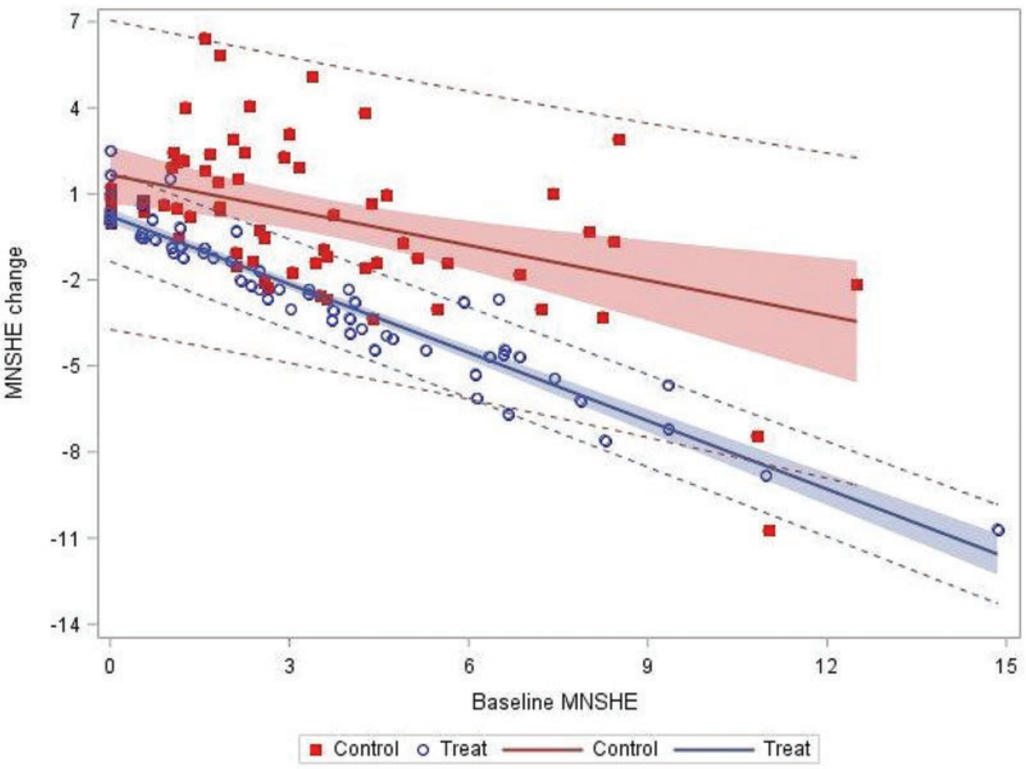


**SUPPLEMENTARY FIG. S2.** Change in MNSHE per week by baseline MNSHE for both intervention and control groups. Solid line: regression line based on Model 1. Shaded area: 95% confidence interval for the regression line. Dotted lines: upper and lower limit of the 95% prediction interval.

Supplementary Table S1. Baseline Characteristics of Participants Included in the Analysis, Compared with the Total Intervention Arm Population

| *Variable* | *Entire population* (n = *76)* | *Analysis population* (n = *70)*[a] |
|---|---|---|
| Age, years | 49.0 (12.2) | 48.7 (12.2) |
| Male, *n* (%) | 38 (50%) | 37 (52.9%) |
| Body mass index, kg/m$^2$ | 26.5 (4.55) | 26.7 (4.59) |
| $HbA_{1c}$, mmol/mol [%] | 61 (9) [7.7 (0.86)] | 61 (9) [7.7 (0.86)] |
| Diabetes duration, years | 28.5 (11.14) | 28.8 (11.14) |
| Duration of CSII therapy, years | 8.8 (6.07) | 8.6 (6.9) |
| Total daily dose, U/kg | 0.573 (0.175) | 0.575 (0.174) |
| Bolus daily dose, U/kg | 0.311 (0.128) | 0.311 (0.127) |
| Gold questionnaire score | 5.2 (1.19) | 5.1 (1.18) |
| Clarke questionnaire score | 4.5 (1.3) | 4.5 (1.3) |

Data are presented as mean (SD).

[a]Six participants from the intervention arm had fewer than 3 days of CGM data at baseline, and they were not included in this analysis. Bosi et al.[S1]

CGM, continuous glucose monitoring; CSII, continuous subcutaneous insulin infusion; SD, standard deviation.

Supplementary Table S2. Univariate Analysis of the Association Between Each Baseline Factor and Reduction of Mean Number of Sensor Hypoglycemic Events (<54 mg/dL) Per Week with the MiniMed 640G System with Suspend Before Low Feature

| *Variable* | P | $R^2$ | *Highly correlated group*[a] | *Model 1* | *Model 2* | *Model 3* |
|---|---|---|---|---|---|---|
| Age | 0.40 | 0.01 | | | | |
| Gender | 0.43 | 0.01 | | | | |
| Region (countries) | 0.49 | 0.05 | | | | |
| Body mass index | 0.88 | 0.0003 | | | | |
| Highest level of education | 0.12 | 0.11 | | X | X | X |
| $HbA_{1c}$ | 0.0006 | 0.16 | | X | X | X |
| Creatinine clearance | 0.9244 | 0.0001 | | | | |
| Most recently used pump model | 0.41 | 0.06 | | | | |
| Primary indication for CSII therapy | 0.93 | 0.03 | | | | |
| Diabetes duration | 0.20 | 0.02 | | X | X | X |
| Duration of CSII therapy | 0.04 | 0.06 | | X | X | X |
| Diabetes-related complications | 0.72 | 0.0002 | | | | |
| Insulin type | 0.78 | 0.02 | | | | |
| Total daily dose (corrected for weight/kg) | 0.06 | 0.05 | A | X | X | X |
| Bolus daily dose (corrected for weight/ kg) | 0.10 | 0.04 | A | | | |
| Percentage of bolus dose | 0.56 | 0.005 | | | | |
| Mean number of boluses per day | 0.43 | 0.009 | | | | |
| Mean number of SMBG per day | 0.30 | 0.02 | | | | |
| Mean SMBG value | 0.0001 | 0.21 | B | | | X |
| SD of SMBG | 0.67 | 0.003 | | | | |
| CV of SMBG | 0.0006 | 0.16 | | X | X | X |
| Gold questionnaire score | 0.81 | 0.0009 | | | | |
| Clarke questionnaire score | 0.68 | 0.003 | | | | |
| Question 3 of Clarke questionnaire | 0.92 | 0.0001 | | | | |
| Question 4 of Clarke questionnaire | 0.54 | 0.006 | | | | |
| MNSHE (<54 mg/dL) per week | 0.0001 | 0.91 | C | X | | |
| MNSHE (<60 mg/dL) per week | 0.0001 | 0.80 | C | X | | |
| MNSHE (<70 mg/dL) per week | 0.0001 | 0.65 | C | X | | |
| Mean duration (minutes) of hypoglycemic events <54 mg/dL | 0.0001 | 0.29 | D | X | | |
| Mean duration (minutes) of hypoglycemic events <60 mg/dL | 0.0001 | 0.30 | D | X | | |
| Mean time (minutes) spent <54 mg/dL | 0.0001 | 0.85 | C | | X | |
| Mean time (minutes) spent <60 mg/dL | 0.0001 | 0.84 | C | | | |
| Mean time (minutes) spent <70 mg/dL | 0.0001 | 0.77 | C | | | |
| AUC <54 mg/dL | 0.0001 | 0.80 | C | | | |
| AUC <60 mg/dL | 0.0001 | 0.83 | C | | | |
| AUC <70 mg/dL | 0.0001 | 0.84 | C | | | |
| MAGE | 0.15 | 0.03 | | X | | |
| Mean SG | 0.0001 | 0.34 | B | X | X | |
| 24-h SD of SG | 0.27 | 0.02 | | | | |
| CV of SG | 0.0001 | 0.39 | | X | X | |

*P* value and $R^2$ are derived from univariate analysis.

[a]Groups of factors highly correlated to each other (Pearson correlation >0.7 or <–0.7 between factors) are denoted with the same letter. "x" indicates that the factor was included in the models shown (Model 1, Model 2, and Model 3) in multivariate analysis.

AUC, area under concentration-time curve; CV, coefficient of variation; MAGE, mean amplitude of glycemic excursions; MNSHE, mean number of sensor hypoglycemic event; SG, sensor glucose; SMBG, self-monitoring of blood glucose.

Supplementary Table S3. Estimated Reduction in Weekly Mean Number of Sensor Hypoglycemic Event <54 mg/dL for Participants with Different Baseline Mean Number of Sensor Hypoglycemic Event <54 mg/dL Based on Model 1

| *Baseline MNSHE* | *Postintervention MNSHE* | *Reduction* | *% of reduction* |
|---|---|---|---|
| 9 | 2.08 | 6.92 | 76.8 |
| 6 | 1.46 | 4.54 | 75.6 |
| 4 | 1.05 | 2.95 | 73.7 |
| 3 | 0.85 | 2.15 | 71.8 |
| 2 | 0.64 | 1.36 | 68.0 |
| 1 | 0.43 | 0.57 | 56.7 |

Supplementary Table S4. Final Multivariate Models (Model 1) of the Association of Baseline Factors and Reduction of Mean Number of Sensor Hypoglycemic Event Per Week Based on Different Thresholds (Sensor Hypoglycemia <54, <60, and <70 mg/dL) in Participants Using the MiniMed 640G with Suspend Before Low Feature Turned on

| *Response* | *Predictive variables* | *Regression coefficient* | *Standard error* | P | $R^2$ (%) |
|---|---|---|---|---|---|
| Reduction in MNSHE <54 mg/dL | BL MNSHE <54 mg/dL | −0.7937 | 0.02979 | 0.0001 | 91.3 |
| Reduction in MNSHE <60 mg/dL | BL MNSHE <60 mg/dL | −0.7290 | 0.0388 | 0.0001 | 83.9 |
| Reduction in MNSHE <70 mg/dL | BL MNSHE <70 mg/dL | −0.6605 | 0.0510 | 0.0001 | 71.2 |

## Supplementary Reference


S1. Bosi E, Choudhary P, de Valk HW, et al.: Efficacy and safety of suspend-before-low insulin pump technology in hypoglycaemia-prone adults with type 1 diabetes (SMILE): an open-label randomised controlled trial. Lancet Diabetes Endocrinol 2019;7:462–472.
